# A functional eEF2K-eEF2 pathway in the NAc is critical for the expression of cocaine-induced psychomotor sensitisation and conditioned place preference

**DOI:** 10.1038/s41398-022-02232-1

**Published:** 2022-11-01

**Authors:** Tehila Beiser, Elvira Lisniansky, Moriya Weitz, Alexey Bingor, Etty Grad, Kobi Rosenblum, Claire Thornton, Rami Yaka

**Affiliations:** 1 Institute for Drug Research (IDR), School of Pharmacy, Faculty of Medicine, The Hebrew University of Jerusalem, Jerusalem, Israel; 2 Sagol Department of Neuroscience, University of Haifa, Haifa, Israel; 3 Department of Comparative Biomedical Sciences, Royal Veterinary College, London, UK

**Keywords:** Molecular neuroscience, Learning and memory

## Abstract

Recent evidence links synaptic plasticity and mRNA translation, via the eukaryotic elongation factor 2 kinase (eEF2K) and its only known substrate, eEF2. However, the involvement of the eEF2 pathway in cocaine-induced neuroadaptations and cocaine-induced behaviours is not known. Knock-in (KI) mice and shRNA were used to globally and specifically reduce eEF2K expression. Cocaine psychomotor sensitization and conditioned place preference were used to evaluate behavioural outcome. Changes in eEF2 phosphorylation were determined by western blot analyses. No effect was observed on the AMPA/NMDA receptor current ratio in the ventral tegmental area, 24 h after cocaine injection in eEF2K-KI mice compared with WT. However, development and expression of cocaine psychomotor sensitization were decreased in KI mice. Phosphorylated eEF2 was decreased one day after psychomotor sensitization and returned to baseline at seven days in the nucleus accumbens (NAc) of WT mice, but not in eEF2K-KI mice. However, one day following cocaine challenge, phosphorylated eEF2 decreased in WT but not KI mice. Importantly, specific targeting of eEF2K expression by shRNA in the NAc decreased cocaine condition place preference. These results suggest that the eEF2 pathway play a role in cocaine-induced locomotor sensitization and conditioned place preference.

## Introduction

In order to find pharmacotherapy for cocaine addiction, it is necessary to understand the neurobiological mechanism underlying craving and relapse. As cocaine relapse may occur after prolonged abstinence, mechanisms resulting in long-lasting modifications in the brain, such as the ones underlying learning and memory processes are required [1]. Even a single cocaine injection can induce neuroadaptations in specific areas of the brain reward system [2, 3]. These changes include enhanced glutamatergic connectivity in striatal projection neurons (SPNs) from the nucleus accumbens (NAc) [2], long-term potentiation (LTP) and long-term depression (LTD) [4, 5].

In the brain, mRNA translation is a critical cellular mechanism, providing tailored responses to the different demands of general homeostasis and synaptic plasticity, and is regulated in both its initiation and elongation phases [6]. Initiation comprises the recruitment of mRNA to the ribosome and is regulated by a family of eukaryotic initiation factors (eIFs [7]). A key element regulating the elongation phase is the eukaryotic elongation factor (eEF2) pathway [8]. eEF2 regulates the translocation of peptidyl-tRNA from the ribosomal A-site to the P-site. eEF2 is itself regulated by inhibitory phosphorylation at Thr56 by an upstream kinase, eEF2 kinase (eEF2K), also known as calmodulin-dependent protein kinase III. Phosphorylated eEF2 prevents peptidyl-tRNA translocation to the ribosome and thus leads to translation inhibition [9–12]. Moreover, regulation of the eEF2 pathway modifies proteome expression in neurons [13]. The eEF2 pathway is involved in neurological processes and diseases such as Alzheimer’s disease, where it was found that amyloid-β oligomers activate eEF2K, leading to decreased synaptic plasticity [14, 15], or in depression and epilepsy, where eEF2K inhibition is related to anti-depressant [16–18] and antiepileptic [19] activity.

Recent studies have identified the impact of cocaine and other drugs of abuse on initiation of translation [20, 21]. Acute and repeated injections of cocaine reduced phosphorylation of eIF2α in the VTA resulting in increased cocaine CPP in both adolescent and adult mice [20]. However, we recently found that the eEF2 (elongation) pathway is affected by dopamine D1 receptor activation in an NMDA receptor-dependent manner [22]. The phosphorylation state of eEF2 is directly linked to synaptic plasticity regulation [23] and learning, and in the context of cue-induced drug seeking, dephosphorylation of both eEF2 and eIF2α occurs. Preventing dephosphorylation (e.g. by inhibition of phosphatases) results in reduced drug seeking [24]. We therefore hypothesized that cocaine-induced neuroadaptations in the NAc and the resulting cocaine-induced behaviours are mediated through the eEF2 pathway.

In the current study, we employed genetically mutated mice that lack eEF2K activity in order to study the involvement of eEF2K in cocaine-induced behaviours. eEF2K knock in (KI) mice, lacking the catalytic unit of eEF2K, have residual eEF2K activity accounting for ~30% phosphorylated (p)eEF2 compared with WT [25]. Following both acute and chronic cocaine exposure, we found decreased neuroadaptations and behaviours in these KI mice, suggesting crucial involvement of eEF2K activity in cocaine-induced behaviours. Furthermore, targeted reduction of eEF2K expression using shRNA lentivirus constructs in the NAc resulted in decreased cocaine conditioned place preference (CPP), implying that eEF2K activity in the NAc alone is play a role for the acquisition of cocaine conditioned reward.

## Materials and methods

### Mice

Knock in (KI) eEF2K KI mice harbour a D273A mutation (on a C57BL/6 background) within the kinase catalytic domain resulting in a reduction of substrate (eEF2) phosphorylation to ~30% of WT; [25] eEF2k itself is expressed normally. To generate the colony, second generation heterozygotes were bred for a minimum of five generations and maintained using standard housing and temperature conditions with 12 h light/dark cycle, in accordance with the guidelines of the Institutional Animal Care Committee of the Hebrew University (Jerusalem, Israel) which also approved the study protocol for animal welfare. The Hebrew University is an AAALAC International accredited institute. Sample sizes were calculated from previously published studies [26, 27]. Male mice were used at age P30-35 (20–26 g), and for all experiments, KI mice were compared with their WT littermates. Mice were excluded from analysis if weight loss of >10% occurred and randomisation carried out where feasible (via Excel). The same investigator analysed the data.

### Electrophysiological recordings

Recordings from the VTA-containing slices were performed as described previously [25]. Electrodes pulled from glass capillaries (3–6 MΩ resistance) were filled with an internal solution containing the following (in mM): 117 CsCH_3_SO_3_, 20 HEPES, 0.4 EGTA, 2.8 NaCl, 5 TEA-Cl, 2.5 Mg_2_ATP, and 0.3 Na_3_GTP, pH 7.2–7.4. Whole-cell currents were recorded in a voltage-clamp configuration using a Multiclamp 700B amplifier (Axon Instruments, Foster City, CA). with a holding potential of +40 mV. Series and input resistance were monitored continuously with a 4 mV depolarizing step given before every afferent stimulus. Experiments were begun only after series resistance had stabilized and data were discarded if series resistance changed by >15%. DA neurons were identified by the presence of Ih-currents [28] tested immediately after break-in, using a series of eight successive hyperpolarizing steps (−10 mV; 250 ms) beginning from an initial holding potential of −50 mV. For AMPAR- and NMDAR-mediated excitatory post synaptic current (EPSCs) recording, a bipolar stainless steel stimulating electrode was placed 100–200 μM rostral to the recording electrode, stimulating afferent fibres at a frequency of 0.1 Hz. To calculate the AMPAR/NMDAR ratio, an average of 12 EPSCs was computed at +40 mV before and after application of AP5 (an NMDAR antagonist, 50 μM, 5 min). The AP5-derived (AMPAR-only) average response was subtracted from that seen in its absence and an average NMDAR-only response was calculated. The peak of the AMPAR-only EPSC was divided by the peak of the NMDAR-only EPSC to give an AMPAR/ NMDAR ratio.

### Psychomotor Sensitization

Locomotor activity was measured in activity boxes (43.2 cm × 43.2 cm × 30 cm) containing 16 photo beam arrays located along each side (Med Associates, St. Albans, VT, USA). The activity was measured by the total beam breaks, which represents horizontal movement. Saline or cocaine treatments were randomly assigned within genotype groups. Following a 3-day acclimation, animals were habituated on the first day to the boxes by placement in the box following a single i.p. injection of saline (1 ml/kg). Over the next 4 days, animals were injected i.p. with either cocaine (15 mg/kg, i.p., standard dose for the protocol [29]) or saline (1 ml/kg), and locomotor activity was measured for 30 min. On the 18th (challenge) day, all animals, including the saline group, received a single i.p. injection of cocaine (15 mg/kg) and locomotor activity was monitored for 30 min [30].

### Conditioned place preference (CPP)

CPP for cocaine was performed as previously described with a preferred cocaine dose (15 mg/kg) commonly used within the field [27, 29, 31]. The CPP apparatus (Med Associates) consists of two visually distinct conditioning compartments (white with wire mesh floor, or black with steel rod floor) connected by a small centre compartment. Infrared beams located at the bottom of the wall allow assessment of animal preference for each compartment. CPP experiments were carried out at predefined times. Following a 3-day acclimation, a biased CPP design was conducted. Mice were placed in the central compartment and allowed free access to all compartments (15 min), from which an initial compartment preference was computed for each mouse. The least preferred compartment was then assigned as the drug-paired compartment. One day after the habituation session, the conditioning period began with mice receiving saline (1 ml/kg, i.p.) or cocaine (15 mg/kg, i.p.) injections on alternating days (for a total of 8 days). Following injection, mice were confined to the allocated compartment for a 15 min period. To evaluate the establishment of cocaine-induced CPP, 1 day following the last conditioning day, mice were placed in the central compartment for 5 min followed by a 15 min period of free access to all compartments. CPP score was calculated as follows:

$$CPP\;score\left( {{{\mathrm{\% }}}} \right) = \frac{{(time\;in\;drug\;paired\;chamber - time\;in\;saline\;paired\;chamber)}}{{total\;time\;spent\;in\;both\;chambers}} \times 100.$$

### Western blot analysis

Following behavioural tests (CPP, PS), NAc tissue was taken from mice for western blot as described previously [26]. Where necessary, samples were pooled to generate sufficient protein. Mice were anesthetized using isoflurane, decapitated and their brains were immediately removed. Coronal sections were prepared, the NAc was microdissected on ice and snap frozen. Tissue was then homogenized (320 mM sucrose, 10 mM Tris-HCl, pH 7.4, 1 mM EDTA, 1 mM EGTA, protease and phosphatase inhibitors [Sigma-Merck, Darmstadt, Germany]) and protein concentration determined (Pierce™ BCA Protein Assay Kit (Pierce, IL, USA)). Samples were denatured (5 min, 95 °C) and 25 or 30 µg protein resolved by 8-12.5% SDS-PAGE. Proteins were transferred to nitrocellulose membrane (Pall Corporation, FL, USA), blocked (5% nonfat dry milk, 1 h, room temperature) and incubated overnight (4 °C) with primary antibodies (1:1000, phospho-eEF2 (Thr56), eEF2 antibodies from Cell Signalling Technology, Danvers, MA). Following washing, membranes were incubated with species-appropriate HRP-conjugated secondary antibody (1:10,000; 1 h, room temperature). Signal was detected and quantified using Clarity™ western ECL and the BioRad ChemiDoc™ XRS + Imaging system (BioRad, CA, USA).

### Stereotactic injections

Lentiviral vectors (hp-EF2K, and scrambled control hp-scr) tagged with GFP were generated by the Center for Gene Manipulation in the Brain, University of Haifa, Haifa, Israel. Male mice (30–40 days) were anesthetized using a mixture of ketamine hydrochloride 100 mg/ml: medetomidine hydrochloride (1 mg/ml): saline (11: 12: 80; 10 µl/g). Mice were also injected s.c. with tramadol hydrochloride solution (Tramal®, Grünenthal, GmbH) for analgesia, diluted to a final dose of 0.05 mg/g. The injections were performed using a Digital Lab Standard™ Stereotactic instrument (Stoelting Co. IL, USA). Mice were injected bilaterally with 1 µL of either the hpEF2K (5 × 10^8^tu/ml) or the hpSCR (1.5 × 10^8^tu/ml) vectors into the NAc (AP (Bregma) +1.2 mm, ML ± 1.0 mm, DV −4.5 mm) at a rate of 0.1 µL/min using a 1 ml syringe equipped with a 32 gauge needle (Hamilton, NA, USA). The incision was sutured and the sedative effects of medetomidine reversed by i.p. injection of atipamezole hydrochloride (50 mg/ml, Eurovet Animal Health B.V, Netherlands). Mice were housed in separate cages for 2 weeks for recovery and for the viral vector to be fully expressed.

### Statistical analysis

All data were assessed for normal distribution. To evaluate significance of two groups, an unpaired *t*-test was used. For multiple comparisons between groups with only one variable, we used one-way ANOVA followed by an appropriate *post hoc* test. For multiple comparisons where there were two variables (e.g. genotype and treatment), data were analysed using a two-way ANOVA (with repeated measures where appropriate) followed by an appropriate *post hoc* test. Data are presented as mean ± S.E.M. The accepted value of significance for all tests was set at *p* < 0.05 and indicated by asterisks in the figures. All group sizes, significant differences and analyses are reported in the figure legends. Individual data points graphed represent the number of experimental units (individual animals). Statistical analysis was performed using the VassarStats website and Graphpad Prism v9. All experiments were reported in accordance with the ARRIVE guidelines [32]. All statistical data analysis are presented in Supplement 1.

## Results

### Acute cocaine exposure increases AMPA/NMDA receptor current ratio in VTA dopamine neurons of eEF2K KI and WT mice

**Fig. 1:**
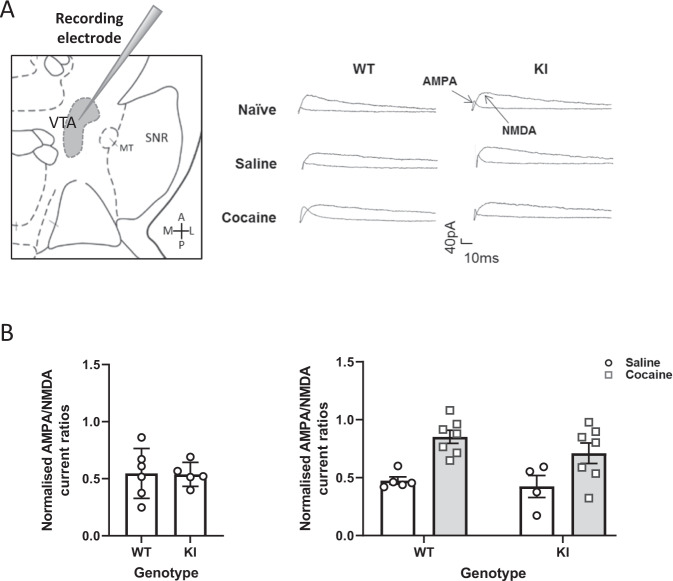
Acute cocaine exposure increases AMPA/NMDA receptor current ratio in the VTA in WT and KI mice. **A** Sample EPSCs were recorded from dopamine neurons in WT and KI naïve mice or WT/KI mice pretreated with cocaine or saline, in the Ventral Tegmental Area (VTA). **B** Peak AMPAR- and NMDAR-mediated EPSCs current ratio from naïve WT and KI mice (*N* = 4–7) expressed as a ratio in the VTA. *Left*: Data were analysed by unpaired *t*-test (*t* = 0.0773), *Right*: Data were analysed by two-way ANOVA. Data expressed as mean ± SEM.

As the response to cocaine has not previously been evaluated in eEF2K KI mice we began our study by characterising their response to acute cocaine injection. Cocaine induces an increase in AMPA/NMDA receptor current ratio in the VTA following a single exposure [3]. Since this alteration required changes in protein synthesis [16], we speculated that the increase in the receptor current ratio will be absent in eEF2K KI mice. Hence, we measured the AMPA/NMDA receptor current ratio in VTA-containing brain slices from naïve mice or 1 day following a single cocaine (15 mg/kg, IP) or saline injection (1 ml/kg, IP; Fig. 1A). Genotype, treatment effects and their interaction were analysed using two-way ANOVA. Although the treatment effect was statistically significant [F (1,19) = 19.06, *p* = 0.0003], the genotype and interaction effects were not significant [F (1,19) = 1.603, *p* = 0.2208; F (1, 19) = 0.3524, *p* = 0.5598], implying that eEF2K KI mice respond in a similar manner to WT to acute cocaine administration (Fig. 1B).

### Decreased cocaine-induced behaviours in eEF2K KI mice

**Fig. 2:**
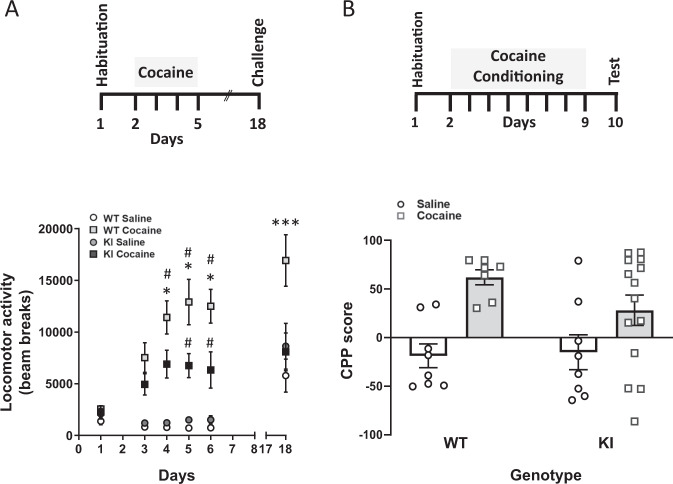
Decreased cocaine-induced behaviours in eEF2K KI mice. For both psychomotor sensitization and condition place preference (CPP), mice were assigned to two groups according to genotype, and then randomly assigned either cocaine (15 mg/kg) or saline (1 ml/kg). **A** No difference was observed in the locomotor activity of either WT or KI mice following saline. Mixed effects model revealed a significant difference for genotype and treatment, [F(3, 29) = 18.82, *P* < 0.0001]. Sidak *post hoc* analysis identified that cocaine increased locomotor activity in both WT and KI mice compared with saline (#*p* < 0.001, days 4–6 for WT, days 5&6 for KI), but cocaine-exposed KI mice demonstrated reduced locomotor activity (*p < 0.05, days 4–6) compared with WT mice. In addition, KI show no expression of psychomotor sensitization following cocaine challenge (15 mg/kg, day 18, *p* > 0.9999, KI cocaine vs KI saline; ***p < 0.0001, WT cocaine vs WT saline; ***p = 0.0001, WT cocaine vs KI cocaine; *N* = 8–10). **B** All mice underwent a standard CPP paradigm for 2 weeks. CPP test was performed for all mice 1 day after the last conditioning session. Values are expressed as mean CPP score ± S.E.M. (*n* = 7–14 per group), analysed by two-way ANOVA.

As there was no effect of eEF2K following acute exposure to cocaine, we therefore explored its role in the subsequent behaviours induced by prolonged exposure to cocaine. We used two classical behavioural paradigms, psychomotor sensitization and conditioned place preference (CPP), to evaluate the effect of eEF2K KI on the neuroadaptations and reward induced by cocaine. Repeated daily injection of cocaine in WT mice induced a gradual increase in locomotor activity representing a normal development phase of sensitization (Fig. 2A). In KI mice, locomotor activity was initially evident however, in agreement with our hypothesis, locomotor activity did not become further elevated (as observed in WT mice) during the course of injections (four daily cocaine injections 15 mg/kg, i.p.; Mixed effects model, F(3, 29) = 18.82, *P* < 0.0001, WT cocaine vs KI cocaine). Following an abstinence of cocaine from day 5 to day 18, all four groups were challenged with a single cocaine dose (15 mg/kg, i.p.). As expected in WT mice, a challenge injection elicited a robust increase in locomotion. However, in eEF2K-KI mice, locomotor activity was comparable with WT and KI saline groups (WT cocaine vs WT saline, *p* < 0.0001; WT cocaine vs KI saline, *p* = 0.0004; WT cocaine vs KI cocaine, *p* = 0.0001, Sidak’s *post-hoc* test).

We next examined the effect of eEF2K-KI on cocaine-conditioned reward using conditioned place preference (CPP) (24). Mice were conditioned with cocaine (15 mg/kg) for 8 days and 1 day following cessation of conditioning, mice were tested for their preferred chamber. Genotype, treatment effects and their interactions were analysed using two-way ANOVA. Although the treatment effect was highly significant [F (1,33) = 14.75, *p* = 0.0005], no interaction nor genotype effects were found ([F (1,33) = 1.356, *p* = 0.2526; F (1,33) = 0.8743, *p* = 0.3566], respectively, Fig. 2B). Although these data suggest that KI mice respond similarly to WT mice in the CPP paradigm, we identified that their responses were much more variable; 4 eEF2K KI mice demonstrated aversion compared with 0 WT mice (Fig. 2B).

### Cocaine reduces phosphorylation of eEF2 in the NAc

**Fig. 3:**
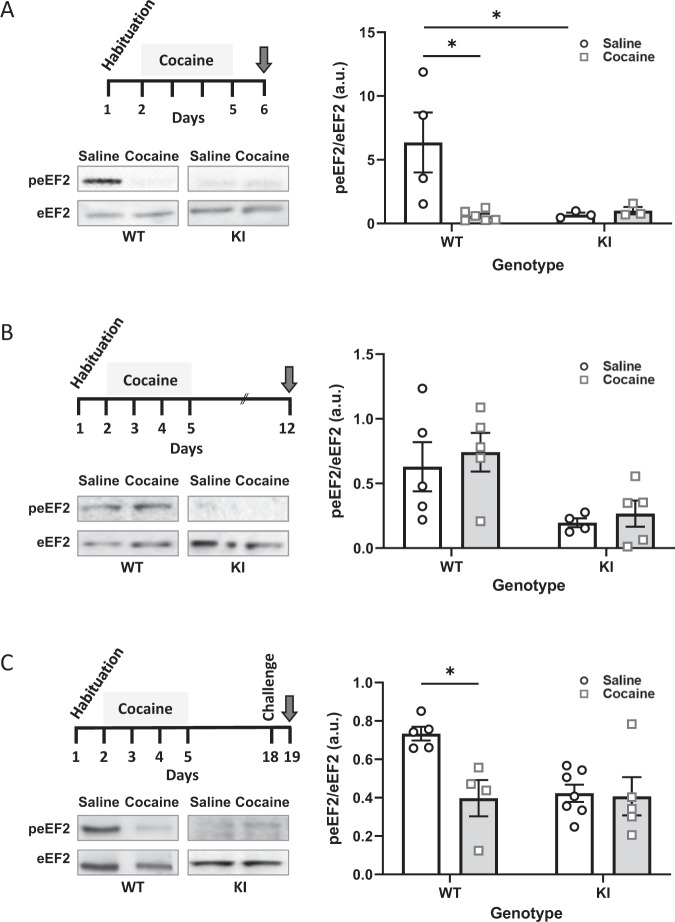
Cocaine reduces phosphorylation of eEF2 in the NAc at 1 day after psychomotor sensitization or following challenge in WT but not KI mice. peEF2/eEF2 ratio analysed by western blot using peEF2 and eEF2 antibodies. NAc brain homogenates were taken from mice euthanized (*N* = 3–7), **A** one day (day 6) following psychomotor sensitization, **B** seven days following psychomotor sensitization. **C** Mice underwent psychomotor sensitization from days 2–5, followed by 14 days of withdrawal and euthanized on day 19, one day following cocaine challenge (15 mg/kg; day 18). Data shown ± SEM. Data were analysed by two-Way ANOVA followed by Tukey’s *post-hoc* where appropriate. *p < 0.05.

Having found that psychomotor sensitization was decreased in the eEF2K KI mice, we speculated that the phosphorylation state of eEF2 (peEF2) in WT mice would be affected accordingly. Therefore, we tested the peEF2 at several time points following repeated cocaine injections (four daily cocaine injections 15 mg/kg, i.p.). A two-way ANOVA revealed a statistically significant interaction between the effects of Genotype and Treatment [F(1,12) = 6.042, *p* = 0.0301]. Simple main effect analysis showed that Genotype effect did not have a statistically significant effect on the phosphorylation state of eEF2 (F1,12 = 4.509, *P* = 0.0052). However, simple main effect analysis showed that cocaine reduced peEF2 significantly (F(1,12) = 4.875, *P* = 0.0475). These results indicate that cocaine significantly decrease the phosphorylation of eEF2 in WT mice but not in KI mice (Fig. 3A). However, when tested 7 days following withdrawal from repeated injections of cocaine, no interaction between genotype and treatment was observed following analysis of eEF2 phosphorylation state (Fig. 3B; interaction between treatment and genotype [F(1,15) = 0.0221, *p* = 0.8838]; Two-way ANOVA). Moreover, when tested 1 day following challenge injection, a 2-Way ANOVA revealed a statistically significant interaction between the effects of Genotype and Treatment [F(1,17) = 5.277, *p* = 0.0346]. Simple main effect analysis showed that both Genotype and Treatment effects did have a statistically significant effect on the phosphorylation state of eEF2 [F(1,17) = 4.656, *P* = 0.0455, F(1,17) = 6.381, *p* = 0.0218, respectively]. These results indicate that cocaine significantly decreases the phosphorylation of eEF2 in WT mice but not in KI mice following cocaine challenge (Fig. 3C). Importantly, in the KI groups, the basal levels of eEF2 phosphorylation was very low, as expected [25], and no significant differences were found between the cocaine and saline groups (Fig. 3A, C).

### Reducing levels of eEF2K in the NAc decreased cocaine CPP

**Fig. 4:**
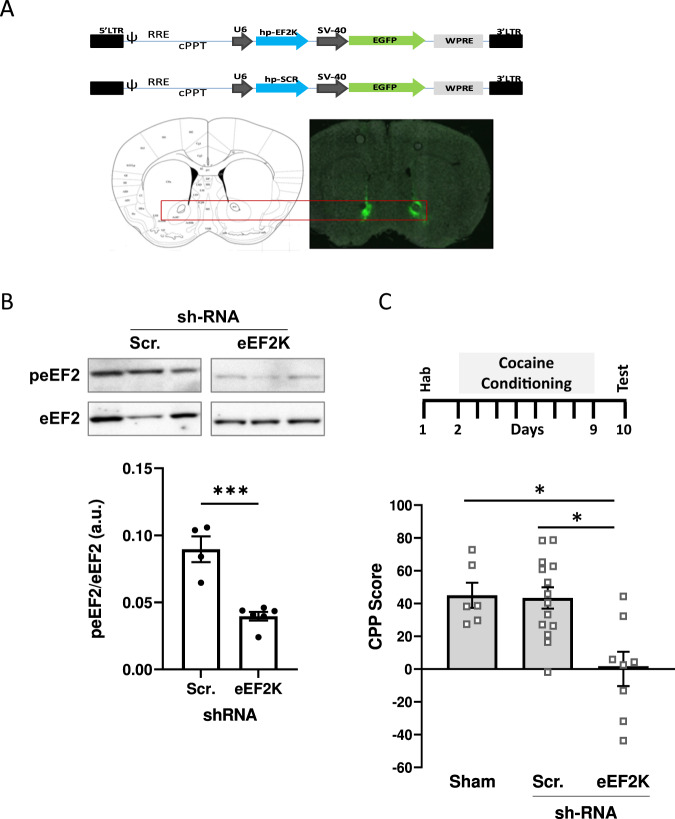
Silencing of eEF2K in the NAc decreased cocaine CPP. Mice were bilaterally microinjected into the NAc with either hp-EF2K – lentiviral vector for eEF2K silencing, or with hp-SCR—lentiviral vector expressing scrambled sequence as a control. **A** A representative coronal section of a mouse brain slice containing the bilateral injection sites at the NAc (GFP, green), 2 weeks after the surgery. **B** Two weeks following injection, NAc protein samples were prepared and analysed with peEF2 and eEF2 antibodies. Histogram depicts the level of peEF2 divided by total eEF2 ± SEM (***p = 0.0004, unpaired *t*-test, *N* = 4–6). **C** Two weeks after viral injection, all mice underwent a standard CPP paradigm. CPP test was performed for all animals 1 day after the last conditioning session. Values are expressed as mean CPP score ± S.E.M. (*n* = 8 per group). Data were analysed by one-way ANOVA followed by Tukey’s *post-hoc* test. *p < 0.05.

We hypothesised that the variability observed following CPP in the eEF2K KI (Fig. 2B) was due to the global nature of the model. To evaluate this hypothesis, we used an alternative strategy, targeting murine eEF2K shRNA specifically to the NAc, a key structure that mediates cocaine rewarding effects. shRNA-eEF2K or scrambled control vector was bilaterally microinjected into the NAc of WT mice and viral GFP expression was subsequently detected after 2 weeks (Fig. 4A). To verify that there was functional integration of the shRNA-eEF2K vector in the NAc, peEF2 levels were determined and found to be significantly decreased compared with scrambled vector (*p* = 0.0004, unpaired t-test; Fig. 4B). To determine the behavioural consequences of specifically silencing eEF2K in the NAc we used the CPP paradigm. eEF2K silencing significantly attenuated the expression of CPP (F(2,25) = 8.799, *p* = 0.0013, one-Way ANOVA). In mice receiving the scrambled control, CPP was established following conditioning, similar to sham animals (*p* = 0.9902, Tukey *post hoc* test). However, the expression of CPP in mice injected with shRNA-eEF2K was significantly decreased (shRNA-eEF2K vs sham; *p* = 0.0073, shRNA-eEF2K vs scrambled shRNA *p* = 0.0017, Fig. 4C), correlating the reduced expression of eEF2K with impairment of the neuroadaptation. Together, these indicate a role of NAc-specific eEF2 pathway in cocaine CPP.

## Discussion

The mechanisms regulating protein translation with respect to brain function in health and disease have been studied extensively, however, this is not the case in cocaine-induced behaviours. In general, reduced peEF2 indicates activation of protein translation and modifications of proteome steady state [8, 11, 33, 34]. When eEF2K is defective, such as in mutated eEF2K KI mice, there is no further change in phospho-eEF2 levels following cocaine administration suggesting that the levels of peEF2 are already too low for change to be detectable or that there is a misregulation of the eEF2 pathway. In either case it suggests that a functional eEF2K is required for cocaine-induced neuroadaptations.

Evidence for the involvement of mRNA translation regulation in psychomotor sensitization has primarily focussed on the initiation phase where both acute and repeated injections of cocaine reduced phosphorylation levels of the initiation factor eIF2α in the VTA [20]. Prolonged LTP from 5 to 40 days was observed in response to acute cocaine injection due to decreased eIF2a phosphorylation, emphasizing its contribution to a process that represents the persistence of cocaine-induced LTP [21]. It was also shown that the regulators of both initiation (eIF2) and elongation (eEF2) were dephosphorylated following cocaine self-administration [24]. Therefore, both phases of protein synthesis are implicated in the regulation of cocaine-induced behaviours. However, our data suggest that eEF2K is not critical in the response to acute cocaine injection.

A link between cocaine-induced LTP, mRNA translation and addictive behaviour was found when translation inhibitors such as anisomycin diminished calcium-permeable AMPAR to saline levels in the NAc of rats trained to self-administer cocaine [35]. We therefore tested the effect of eEF2K impairment during chronic cocaine exposure and its behavioural consequences; we used both psychomotor sensitization and CPP paradigms, comparing the behaviour of eEF2K KI with WT mice. In cocaine-induced psychomotor sensitization, development and expression of sensitisation was decreased in eEF2K KI mice. However, specific targeting of eEF2K in the NAc through injection of shRNA, rather than global KI, was required to abolish CPP expression.

The results obtained from two different behavioural paradigms, psychomotor sensitization and CPP, confirmed our hypothesis that eEF2K is essential for the expression of these behaviours. Moreover, we found that these behaviours are correlated with changes in the phosphorylation of eEF2 in the NAc. Following the development phase of psychomotor sensitization in WT mice, we found dephosphorylation of eEF2 (Fig. 2). Prolonged withdrawal from cocaine injections (without any cue, context exposure or drug injection) resulted in a return of eEF2 phosphorylation to baseline. However, following cocaine challenge after prolonged withdrawal, a significant decrease in peEF2 levels was again apparent, coupled with psychomotor sensitisation, which was absent in the parallel experiments in KI mice. The behavioural neuroadaptation therefore required an immediate, rather than a prolonged, adaptive mRNA translation, which primed the system for subsequent cocaine challenge. This challenge resulted in reduction of peEF2 suggesting a reinitiation of the dephosphorylation-rephosphorylation cycle.

Dephosphorylation of eEF2 results in promotion of the elongation phase of protein synthesis [8]. Previously, increased protein translation via eEF2 and eIF2 dephosphorylation was observed during cue-induced cocaine seeking [24]. We also observed decreased eEF2 phosphorylation correlating with cocaine-mediated synaptic plasticity suggesting that protein targets which underpin the machinery of learning and memory may alter their expression. Such targets may include CaMKIIα, p70S6K and GluA1 [36], however more recently, profound and widespread cocaine-induced changes in the proteome, both specifically in the NAc and more widely throughout the brain, have been identified [37, 38]. One limitation of our study (and that of the field in general) is that research has focused on responses in male mice. The recent proteomics studies, however, make it clear that there are significant sex-specific differences in the response to cocaine exposure [38]. Future work focused on deriving the mechanistic differences between the male and female responses is critical to determine clinically relevant interventions for male and female users of cocaine.

The NAc is critical for the expression of addictive behaviours and the eEF2K KI mouse model results in a global reduction in eEF2K activity. Therefore, we specifically targeted eEF2K in the NAc using shRNA, resulting in a reduction of peEF2 to levels similar to those observed in the KI mouse. Even though the reduction of expression was limited to the NAc, expression of CPP was abolished, suggesting that eEF2 pathway in the NAc is necessary and sufficient for the expression of cocaine-mediated CPP. In summary, we have demonstrated that cocaine modulates the regulation of protein translation via the eEF2 pathway.

## Supplementary information

Supplement 1
