# Supplementary material for: A functional eEF2K-eEF2 pathway in the NAc is critical for the expression of cocaine-induced psychomotor sensitisation and conditioned place preference
Source: Transl Psychiatry. 2022 Nov 1;12:460. doi: 10.1038/s41398-022-02232-1 (PMC9626485; doi:10.1038/s41398-022-02232-1)
Supplement: Supplementary file 1 — Supplement 1 [file 41398_2022_2232_MOESM1_ESM.docx]

| **Figure** | **Test** |  | **F value** | **DF** | **p-value** |
| --- | --- | --- | --- | --- | --- |
| **1B, left** | Unpaired t test |  | *t=0.0773 | 9 | 0.9401 |
| **1B, right** | 2-Way ANOVA  followed by Sidak’s post-hoc | Interaction | 0.3524 | 1, 19 | 0.5598 |
|  |  | Genotype | 1.603 | 1, 19 | 0.2208 |
|  |  | Treatment | 19.06 | 1, 19 | 0.0003 |
| **2A** | Mixed-effects model, followed by Sidak’s post-hoc | Time | 21.45 | 5, 141 | <0.0001 |
|  |  | Genotype | 18.82 | 3, 29 | <0.0001 |
|  |  | Time x Genotype | 3.785 | 15, 141 | <0.0001 |
| **2B** | 2-Way ANOVA  followed by Tukey's post-hoc | Interaction | 1.356 | 1, 33 | 0.2526 |
|  |  | Genotype | 0.8743 | 1, 33 | 0.3566 |
|  |  | Treatment | 14.75 | 1, 33 | 0.0005 |
| **3A** | 2-Way ANOVA  followed by Tukey's post-hoc | Interaction | 6.042 | 1, 12 | 0.0301 |
|  |  | Genotype | 4.509 | 1, 12 | 0.0552 |
|  |  | Treatment | 4.875 | 1, 12 | 0.0475 |
|  |  |  |  |  |  |
| **3B** | 2-Way ANOVA  followed by Tukey's post-hoc | Interaction | 0.02211 | 1, 15 | 0.8838 |
|  |  | Genotype | 10.52 | 1, 15 | 0.0055 |
|  |  | Treatment | 0.4258 | 1, 15 | 0.5239 |
| **3C** | 2-Way ANOVA  followed by Tukey's post-hoc | Interaction | 5.277 | 1, 17 | 0.0346 |
|  |  | Genotype | 4.656 | 1, 17 | 0.0455 |
|  |  | Treatment | 6.381 | 1, 17 | 0.0218 |
| **4B** | Unpaired t test |  | *t=5.777 | 8 | 0.0004 |
| **4C** | Ordinary One-Way ANOVA,  followed by Tukey's post-hoc | Treatment | 8.799 | 2, 25 | 0.0013 |

Supplement 1: Beiser et al.,

**Table 1:**

**Multiple comparisons:**

**Fig. 1B (right panel)**

| Šídák's multiple comparisons test | Summary | Adjusted P Value |
| --- | --- | --- |
|  |  |  |
| WT:Saline vs. WT:Cocaine | * | 0.0106 |
| WT:Saline vs. KI:Saline | ns | 0.9988 |
| WT:Saline vs. KI:Cocaine | ns | 0.1925 |
| WT:Cocaine vs. KI:Saline | ** | 0.0064 |
| WT:Cocaine vs. KI:Cocaine | ns | 0.6284 |
| KI:Saline vs. KI:Cocaine | ns | 0.1050 |

**Fig. 2A**

| Šídák's multiple comparisons test | Summary | Adjusted P Value |
| --- | --- | --- |
|  |  |  |
| Row 2 |  |  |
| WT Saline vs. WT Cocaine | ns | >0.9999 |
| WT Saline vs. KI Saline | ns | >0.9999 |
| WT Saline vs. KI Cocaine | ns | >0.9999 |
| WT Cocaine vs. KI Saline | ns | >0.9999 |
| WT Cocaine vs. KI Cocaine | ns | >0.9999 |
| KI Saline vs. KI Cocaine | ns | >0.9999 |
|  |  |  |
| Row 3 |  |  |
| WT Saline vs. WT Cocaine | * | 0.0459 |
| WT Saline vs. KI Saline | ns | >0.9999 |
| WT Saline vs. KI Cocaine | ns | 0.8154 |
| WT Cocaine vs. KI Saline | * | 0.0253 |
| WT Cocaine vs. KI Cocaine | ns | 0.9982 |
| KI Saline vs. KI Cocaine | ns | 0.7925 |
|  |  |  |
| Row 4 |  |  |
| WT Saline vs. WT Cocaine | **** | <0.0001 |
| WT Saline vs. KI Saline | ns | >0.9999 |
| WT Saline vs. KI Cocaine | ns | 0.1092 |
| WT Cocaine vs. KI Saline | **** | <0.0001 |
| WT Cocaine vs. KI Cocaine | ns | 0.4168 |
| KI Saline vs. KI Cocaine | ns | 0.0794 |
|  |  |  |
| Row 5 |  |  |
| WT Saline vs. WT Cocaine | **** | <0.0001 |
| WT Saline vs. KI Saline | ns | >0.9999 |
| WT Saline vs. KI Cocaine | ns | 0.1243 |
| WT Cocaine vs. KI Saline | **** | <0.0001 |
| WT Cocaine vs. KI Cocaine | * | 0.0356 |
| KI Saline vs. KI Cocaine | ns | 0.1556 |
|  |  |  |
| Row 6 |  |  |
| WT Saline vs. WT Cocaine | **** | <0.0001 |
| WT Saline vs. KI Saline | ns | >0.9999 |
| WT Saline vs. KI Cocaine | ns | 0.2244 |
| WT Cocaine vs. KI Saline | **** | <0.0001 |
| WT Cocaine vs. KI Cocaine | * | 0.0345 |
| KI Saline vs. KI Cocaine | ns | 0.2875 |
|  |  |  |
| Row 7 |  |  |
| WT Saline vs. WT Cocaine | **** | <0.0001 |
| WT Saline vs. KI Saline | ns | 0.9986 |
| WT Saline vs. KI Cocaine | ns | >0.9999 |
| WT Cocaine vs. KI Saline | *** | 0.0004 |
| WT Cocaine vs. KI Cocaine | *** | 0.0001 |
| KI Saline vs. KI Cocaine | ns | >0.9999 |

**Fig. 2B**

| Tukey's multiple comparisons test | Summary | Adjusted P Value |
| --- | --- | --- |
|  |  |  |
| WT:Saline vs. WT:Cocaine | * | 0.0120 |
| WT:Saline vs. KI:Saline | ns | 0.9986 |
| WT:Saline vs. KI:Cocaine | ns | 0.1352 |
| WT:Cocaine vs. KI:Saline | * | 0.0176 |
| WT:Cocaine vs. KI:Cocaine | ns | 0.4231 |
| KI:Saline vs. KI:Cocaine | ns | 0.1882 |

**Fig. 3A**

| Tukey's multiple comparisons test | Summary | Adjusted P Value |
| --- | --- | --- |
|  |  |  |
| WT:Saline vs. WT:Cocaine | * | 0.0125 |
| WT:Saline vs. KI:Saline | * | 0.0384 |
| WT:Saline vs. KI:Cocaine | ns | 0.0516 |
| WT:Cocaine vs. KI:Saline | ns | >0.9999 |
| WT:Cocaine vs. KI:Cocaine | ns | 0.9945 |
| KI:Saline vs. KI:Cocaine | ns | 0.9985 |

**Fig. 3B**

| Tukey's multiple comparisons test | Summary | Adjusted P Value |
| --- | --- | --- |
|  |  |  |
| WT:Saline vs. WT:Cocaine | ns | 0.9354 |
| WT:Saline vs. KI:Saline | ns | 0.1894 |
| WT:Saline vs. KI:Cocaine | ns | 0.2739 |
| WT:Cocaine vs. KI:Saline | ns | 0.0731 |
| WT:Cocaine vs. KI:Cocaine | ns | 0.1056 |
| KI:Saline vs. KI:Cocaine | ns | 0.9852 |

**Fig. 3C**

| Tukey's multiple comparisons test | Summary | Adjusted P Value |
| --- | --- | --- |
|  |  |  |
| WT:Saline vs. WT:Cocaine | * | 0.0244 |
| WT:Saline vs. KI:Saline | * | 0.0167 |
| WT:Saline vs. KI:Cocaine | * | 0.0201 |
| WT:Cocaine vs. KI:Saline | ns | 0.9935 |
| WT:Cocaine vs. KI:Cocaine | ns | 0.9997 |
| KI:Saline vs. KI:Cocaine | ns | 0.9981 |

**Fig. 4C**

| Tukey's multiple comparisons test | Summary | Adjusted P Value |
| --- | --- | --- |
|  |  |  |
| Ctrl vs. Scrambled | ns | 0.9902 |
| Ctrl vs. sh-eEf2K | ** | 0.0073 |
| Scrambled vs. sh-eEf2K | ** | 0.0017 |
|  |  |  |
|  |  |  |
|  |  |  |
